# Concurrent femoral neck fractures following pelvic irradiation: a case report

**DOI:** 10.1186/1752-1947-3-9332

**Published:** 2009-12-16

**Authors:** Raphael Omotayo Ayorinde, Clement Abu Okolo

**Affiliations:** 1 Department of Orthopaedic and Trauma, University College Hospital, Ibadan, Nigeria; 2 Department of Pathology, University of Ibadan/University College Hospital, Ibadan, Nigeria

## Abstract

**Introduction:**

Fracture of the neck of the femur is common in older people. It often occurs in a single hip, with osteoporosis being the most common cause. Sometimes this fracture may also occur following pelvic irradiation, though this is not common. To the best of our knowledge, we present the first reported case in Nigeria of concurrent bilateral fractures of the femoral neck following pelvic irradiation.

**Case presentation:**

A 74-year-old Nigerian woman presented at our surgical outpatients department with a 5-month history of pain in both hips and a 4-month history of inability to walk. She had had pelvic irradiation for carcinoma of the cervix 2 years earlier. Pelvic radiographs confirmed bilateral subcapital neck fractures.

**Conclusion:**

Patients with hip pain who have been treated with pelvic irradiation should be thoroughly investigated for hip fractures.

## Introduction

Hip fractures are common in older people and are a major source of morbidity and mortality, especially in women. Most of these fractures are related to osteoporosis and are often precipitated by trivial injuries. However, it is well documented that therapeutic radiation can result in bone damage and may increase the risk of fracture [1]. Magnetic resonance imaging and standard radiographs of the hips are useful diagnostic tools [2]. We report a case of bilateral femoral neck fractures presenting after radiotherapy for a gynaecological malignancy.

## Case presentation

A 74-year-old Nigerian woman was referred to our surgical outpatients department from the Radiotherapy Department in September 2007 with a 5-month history of pain in both hips and a 4-month history of inability to walk. The pain had started in the right hip and involved the left hip about a month later. The pain was insidious and was associated with a worsening limp, which required her to walk with the aid of a walking stick. She had been bedridden for about 4 months before presentation but she had no history of falls.

**Figure 1:**
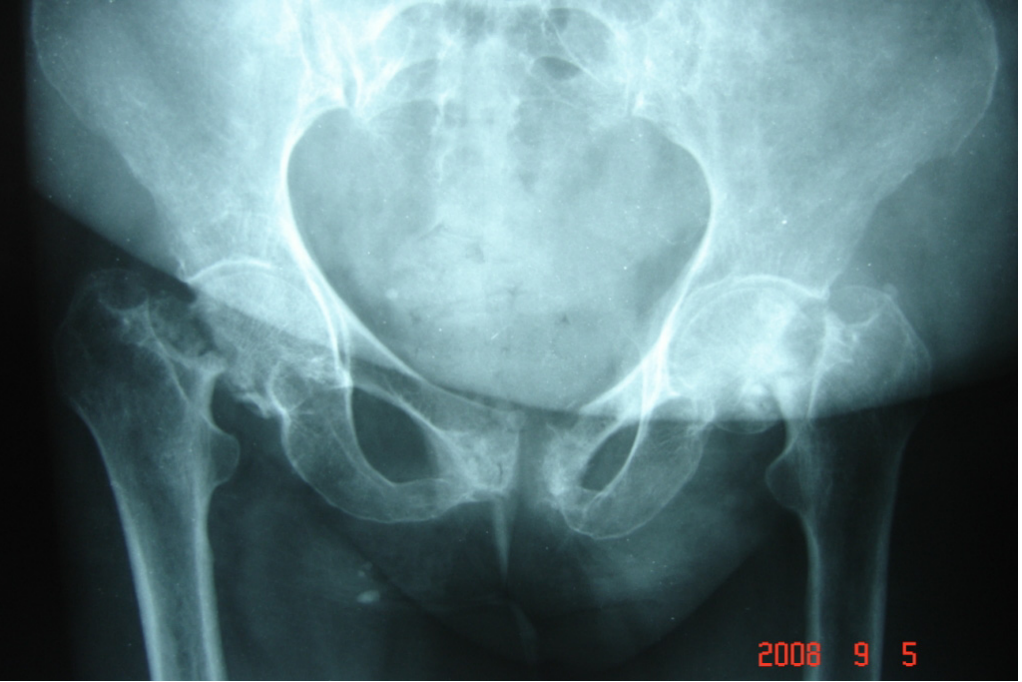
**Pre-operative antero-posterior radiograph**.

She had been diagnosed with a stage 1b invasive squamous cell carcinoma of the cervix 2 years before presentation and was treated primarily with 45Gy of external radiation therapy for 42 days in fractionated doses and had been symptom-free thereafter. A pelvic radiograph showed subcapital fractures of both femurs, with osteonecrosis of the heads (Figure 1).

**Figure 2:**
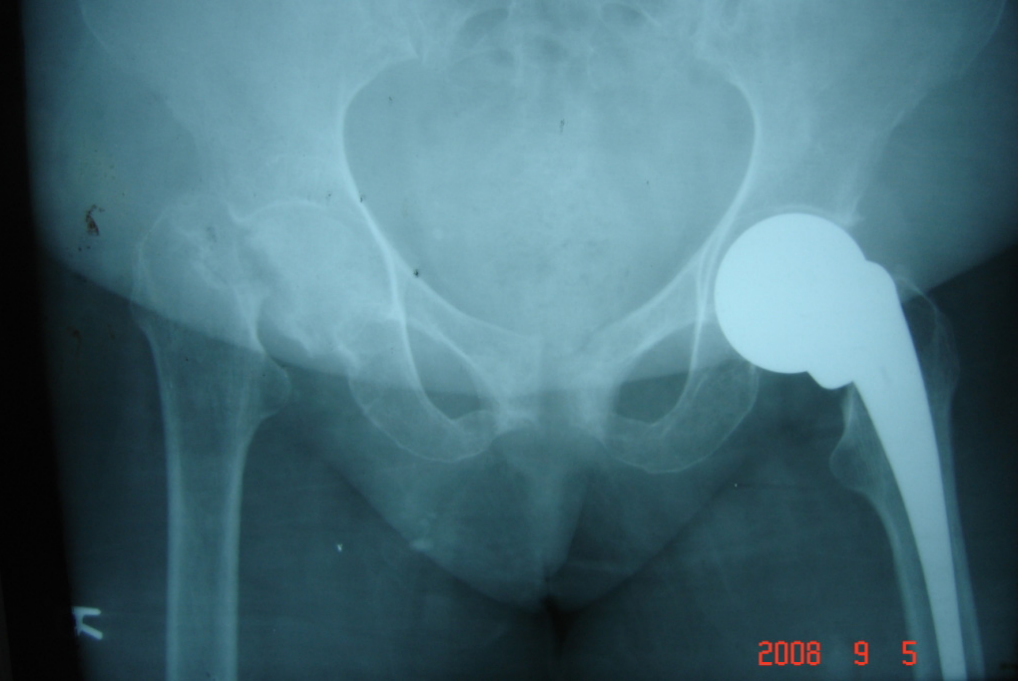
**Pelvic radiograph after left hip hemiarthroplasty**.

**Figure 3:**
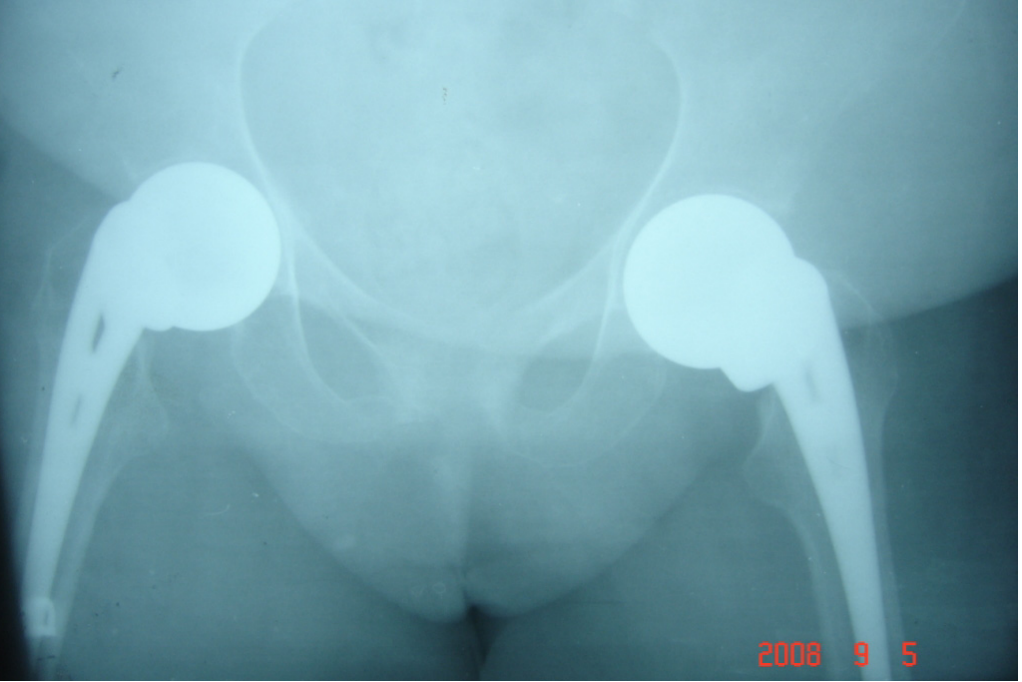
**Pelvic radiograph after bilateral hip hemiarthroplasty**.

She underwent bilateral hemiarthroplasty with non-cemented Austin Moore stems at 8-week intervals (Figures 2 and 3). The histology showed osteoporosis and necrosis, but no malignant cells. The patient is now pain free and walks with a Zimmer frame.

## Discussion

Radiotherapy is the mainstay of treatment of advanced female pelvic malignancies, either in curative or palliative settings [3]. The bony structures of the pelvis and groin lie in close proximity to the genitourinary pelvic organs, gastrointestinal pelvic organs, and the lymphatic drainage of these organs. Therefore, during irradiation, there is an associated high rate of morbidity related to pelvic organs. However, damage to the bones is rarely taken into account, because they are relatively radioresistant. The main evidence of the effect of irradiation on fracture risk comes from a long-term follow-up study of two European randomised trials (Stockholm I and II) evaluating the effect of short-course irradiation in patients with operable rectal cancer [4,5]. It was established that the patients who underwent short-course irradiation were twice as likely to be admitted to hospital with hip fractures as patients who did not undergo irradiation.

Bilateral femoral neck fractures have also been reported in the literature as resulting from long-term steroid therapy, chronic renal insufficiency, in patients with abnormal anatomy of the neck of the femur and as stress fractures in young athletes [6-9]. Our patient, however, did not match any of these conditions.

A search through the medical records in our hospital and in our regional journals showed no patient presenting with hip fracture following pelvic irradiation. This may indicate that African blacks are less likely to develop pelvic fractures following irradiation, although Nancy *et al*. [1] did not find any statistical interaction between race and irradiation therapy. Another reason could be that many of our patients who had been treated may not have lived long enough to develop radiation-induced fractures of the hip. The late presentation and diagnosis of the hip fractures in this patient is worthy of note. The patient presented with hip pain, for which she was treated with analgesics only; no radiological investigation was done until the patient was bed ridden. The rarity of this condition in our hospital may have been responsible for the low index of suspicion. The presentation of this patient fits into the classical symptom and signs suggested by LaVelle, "prodromal pain localised to the hip or radiating to the knee, followed by a gradually increasing limp and disability, as might occur with a slipped femoral epiphysis. Although the patient is able to walk, coxa vara deformity may have already occurred" [10]. Hip pain in patients treated with pelvic irradiation should make the physician suspect radiation-induced insufficiency fractures of the pelvis. In the study by Feltl *et al*. [3], all patients with symptomatic pelvic bone fractures had pain as the first symptom. The roentgenographic characteristics are well described by Stephenson and Cohen [11] and suggest that diagnosis of these fractures could be made on plain X-ray before complete fractures occurred. Computed tomography and magnetic resonance imaging [12] are also useful in evaluating hip pain in patients treated with pelvic irradiation.

## Conclusion

African blacks are predisposed to hip fractures following pelvic irradiation. Hip pain in patients who have had pelvic irradiation should therefore be thoroughly investigated for hip fractures. It should also be noted that complications following irradiation may present months or years later.

## Consent

Written informed consent was obtained from the patient for publication of this case report and any accompanying images. A copy of the written consent is available for review by the Editor-in-Chief of this journal.

## Competing interests

The authors declare that they have no competing interests.

## Authors' contributions

ROA managed the patient, conceptualised the write up, and wrote the initial draft of the manuscript. CAO performed the histological examination of the excised heads, and was a major contributor to the manuscript, especially the histological results. Both authors read and approved the final manuscript.
